# The complete chloroplast genome of *Asarum heterotropoides* Fr. Schmidt var. *mandshuricum* (Maxim.) Kitag. (Aristolochiaceae), a traditional Chinese medicine herb

**DOI:** 10.1080/23802359.2021.1938718

**Published:** 2021-08-04

**Authors:** Yingxin Sun, Qiang Ai, Zeliang Lü, Cuijing Liu

**Affiliations:** Cultivation Base of State Key Laboratory for Ecological Restoration and Ecosystem Management, College of Chinese Medicine Materials, Jilin Agricultural University, Changchun, China

**Keywords:** Chloroplast genome, Aristolochiaceae, phylogenetic analysis

## Abstract

The complete chloroplast genome of *A. heterotropoides* var. *mandshuricum* reported herein was a circular DNA molecule of 160,262 bp in length. The typical quadripartite structure of the genome consisted of a pair of inverted repeats (IRs) of 27,262 bp separated by a large single-copy (LSC) region of 88,927 bp and a small single-copy region (SSC) of 16,811 bp. The overall GC content of the genome is 38.45%, with 36.7%, 33.1%, and 43.0% in LSC, SSC, IR regions, respectively. The cp genome encoded 125 genes, including 83 protein-coding genes, 34 tRNA genes, and 8 rRNA genes. 138 SSRs were identified in the genome. Phylogenetic anlysis showed the position of *A. heterotropoides* var. *mandshuricum* is closely related to A. heterotropoides.

*Asarum* L., a type genus of Aristolochiaceae, which is mainly found in northern temperate distribution, contains about 100 species (Sinn et al. 2015). *Asarum heterotropoides* Fr. Schmidt var. *mandshuricum* (Maxim.) Kitag., also known as ‘xixin’, which is a perennial herb spread in northeast of China, is one of the three original plants used as Traditional Chinese Medicine(TCM) in *Asarum* L. Due to the high similarity of the three species of *Asarum* L. applied to TCM, it is difficult to distinguish them morphologically. Since chloroplast genome has been widely utilized for reconstructing phylogenetic relationships and development of DNA barcodes and molecular markers for the identification of plant species (Huang et al. 2020), it is more accurate to search for differences at chloroplast genome. Therefore, we assembled the complete chloroplast genome of *A. heterotropoides* var. *mandshuricum* using *NGS* technology. The validated complete chloroplast genome sequence was submitted to GenBank with accession number MW526993.

Fresh leaves of *A. heterotropoides* var. *mandshuricum* were collected from Jilin Agricultural University Medicinal Herbs Garden (Jilin, China, 43°48′25″N, 125°24′30″E). A specimen was deposited at Herbarium of Jilin Agricultural University (Zeliang Lü, email: lvzeliang@foxmail.com) under the voucher number 200815. The total genomic DNA was extracted with the TruSeq DNA sample Preparation kit (Vanzyme, China). The chloroplast genomic library was constructed with PCR technology and genomic paired-end (PE150) sequencing was performed on an Illumina Hiseq 2500 platform. A total of 2.0 Gb high-quality reads were obtained after trimming with FastQC v0.11.8 (Chen et al. 2018). Chloroplast genome was assembled using metaSPAdes v3.13.0 (Nurk et al. 2017) and annotated in CPGAVAS2 (Shi et al. 2019) with the chloroplast genome of *A. heterotropoides* (MK577409) chloroplast as the reference sequence.

The complete cp genome of *A. heterotropoides* var. *mandshuricum* is a typical circular quadripartite structure of 160,262 bp in length, consisting of a pair of inverted repeats (IRs) of 27,262 bp separated by a large single-copy (LSC) region of 88,927 bp and a small single-copy region (SSC) of 16,811 bp. The overall GC content of the genome is 38.45%, with 36.7%, 33.1%, and 43.0% in LSC, SSC and IR regions, respectively. The cp genome encoded 125 unique genes, including 83 protein-coding genes, 34 tRNA genes, and 8 rRNA genes. Among these, 9 protein-coding genes (*rpl23*, *rps19*, *rps7*, *ycf15*, *rps12*, *ndhB*, *rpl2*, *ycf1* and *ycf2*), 7 tRNA genes (*trnA-UGC*, *trnL-CAA*, *trnM-CAU*, *trnN-GUU*, *trnR-ACG*, *trnV-GAC* and *trnE-UUC*) and 4 rRNA genes (*rrn16*, *rrn23*, *rrn4.5* and *rrn5*) were found duplicated in the IR regions, respectively. 15 genes (*trnK-UUU*, *trnS-CGA*, *atpF*, *rpoC1*, *trnL-UAA*, *trnV*, *rps12*, *ndhB*, *trnA-UGC*, *ndhA*, *ycf1*, *trnE-UUC*, *ycf15*, *ycf2* and *rpl2*) have one intron and 1 gene (*ycf3*) harbor two introns.

The total of 138 simple sequence repeats (SSRs) were identified in *A. heterotropoides* var. *mandshuricum* chloroplast genome that can be classified into 78 mononucleotide, 28 dinucleotide, 6 trinucleotide, 10 tetranucleotide, 10 pentanucleotide and 6 hexanucleotide using a Perl script MISA (Beier et al. 2017). Mononucleotide repeats were the largest kind of repetition in a number of SSRs and high A/T ratios were observed in the SSRs in this genome.

The phylogenetic analysis was generated based on the complete cp genome of *A. heterotropoides* var. *mandshuricum* and other 10 species within Aristolochiaceae. We constructed a maximum likelihood (ML) tree using MEGA with the combined rapid bootstrap (1000 replicates) after the sequences were aligned using MAFFT v7.475 (Katoh and Standley 2013). Phylogenetic position indicated that *A. heterotropoides* var. *mandshuricum* was closed to the *A. heterotropoides*, and formed another strongly supported monophyletic group with *Saruma henryi* (Figure 1). Besides, the results of this study are similar to previous study (Li et al. 2019).

**Figure 1.**
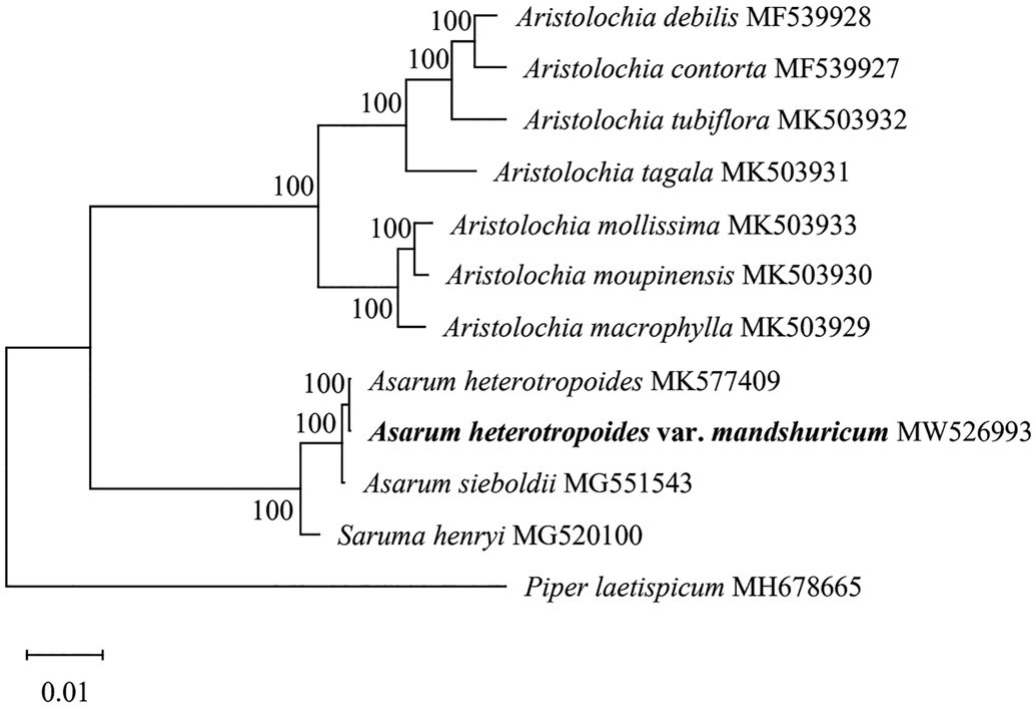
The ML tree based on the cp genome of *A. heterotropoides* var. *mandshuricum* and other 11 species that download from Genbank and *Piper laetispicum* as the outgroup. The numbers on the branches are bootstrap values. *A. heterotropoides* var. *mandshuricum* is in bold.

## Data Availability

The genome sequence data that support the findings of this study are openly available in GenBank of NCBI at (https://www.ncbi.nlm.nih.gov/) under the accession number MW526993. The associated BioProject, SRA, and Bio-Sample numbers are PRJNA727527, SRR14433893, and SAMN19022981 respectively.
